# A Case of Successful Perioperative Rehabilitation in Two-Stage Revision Total Hip Arthroplasty for an Intraoperative Acetabular Fracture: Insights Into Interim-Period Rehabilitation Strategies

**DOI:** 10.7759/cureus.78619

**Published:** 2025-02-06

**Authors:** Shusuke Nojiri, Yusuke Osawa, Shinya Tanaka, Yasuhiko Takegami

**Affiliations:** 1 Department of Rehabilitation, Nagoya University Hospital, Nagoya, JPN; 2 Department of Orthopaedic Surgery, Nagoya University Graduate School of Medicine, Nagoya, JPN

**Keywords:** functional recovery, gait acquisition, muscle strength, physical function, prosthesis-free interval, revision arthroplasty, two-stage exchange

## Abstract

Two-stage revision arthroplasty often results in poor functional outcomes. Rehabilitation strategies to maximize functional recovery after two-stage revision arthroplasty have not yet been established. This report presents a case of successful rehabilitation in two-stage revision total hip arthroplasty (THA). A 75-year-old Japanese woman underwent primary THA and experienced an intraoperative acetabular fracture. Staged revision THA was performed because of a large bone defect. After the first-stage implant removal, progressive muscle strength training, such as quadriceps and isometric exercises for the hip muscles, was performed with consideration of bone fusion, in addition to strengthening the unaffected limbs. During the interim period, an improvement in muscle strength was observed in both the upper and lower limbs. After the second-stage reimplantation, weight-bearing was gradually allowed. Three weeks after full weight-bearing was allowed, the patient was able to walk at 0.67 m/s with a cane. Further recovery of walking speed was achieved after a further four weeks of inpatient rehabilitation, reaching 0.90 m/s. In this case, interim-period muscle strength training was assumed to have contributed to early gait acquisition after reimplantation, without interfering with bony fusion. Well-worked muscle strength training to maintain or even improve muscle strength during the prosthesis-free interval may be important for functional recovery after two-stage revision arthroplasty.

## Introduction

Total hip arthroplasty (THA) is an effective treatment for reducing pain and improving functional mobility in those with hip dysfunction typified by osteoarthritis. However, some THA-related complications sometimes result in poor outcomes, including functional mobility and quality of life. Intraoperative acetabular fracture with pelvic discontinuity is a rare, but gradually increasing complication of primary THA [1-3], and is associated with poor outcomes [4].

Two-stage revision arthroplasty is relatively common as a treatment for periprosthetic joint infection (PJI), which consists of first-stage implant removal and second-stage reimplantation with a six-eight-week interval. In cases of two-stage revision THA, insufficient recovery of muscle and gait function related to immobilization and disuse during the interim period is often observed [5,6], as non- or at best partial weight-bearing ambulation is common until reimplantation [7-9]. Although muscle and gait functions are key outcomes in those with musculoskeletal disorders, perioperative rehabilitation strategies to maximize functional recovery in two-stage revision arthroplasty have not been well-established.

It is often difficult for therapists to prescribe exercise programs, particularly strength training, in the rehabilitation of patients with fractures, because of the need to achieve bone fusion. This can be even more challenging in the interim period of two-stage revision THA with acetabular fracture, where acetabular bone fusion must be achieved while minimizing the adverse effects of immobility and disuse. Here, we report a case of successful rehabilitation in a two-stage revision THA for an intraoperative acetabular fracture during primary THA.

## Case presentation

Patient information

**Figure 1:**
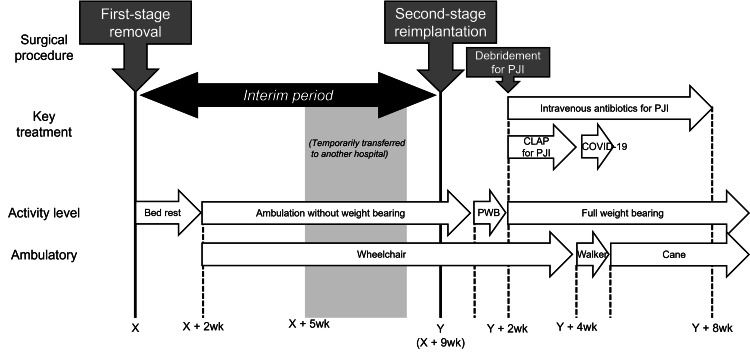
Overview of clinical course CLAP, continuous local antibiotic perfusion; PJI, periprosthetic joint infection; PWB, partial weight bearing; THA, total hip arthroplasty

**Figure 2:**
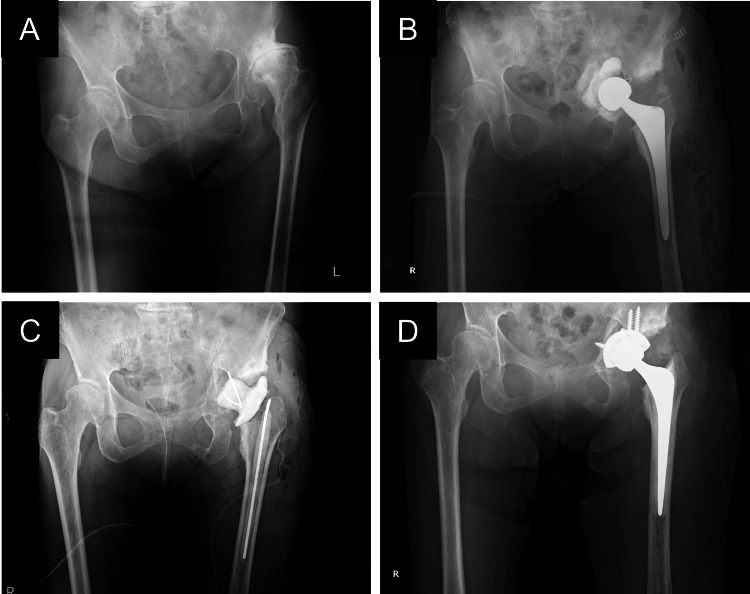
X-ray images of the hip A, before primary THA; B, before first-stage removal; C, after first-stage removal; D, after second-stage reimplantation

An overview of the patient’s clinical course is presented in Figure 1. A 75-year-old Japanese woman (height, 147.0 cm; body weight, 41.3 kg) has suffered from hip pain on the left due to hip osteoarthritis (Figure 2A) and underwent primary THA using a cemented cup at the referring hospital. Preoperative status included full independence in activities of daily living (ADL) and aerobic exercise in the swimming pool three times a week. During the operation, the acetabulum fractured accidentally, and the implant perforated the pelvis (Figure 2B).

**Figure 3:**
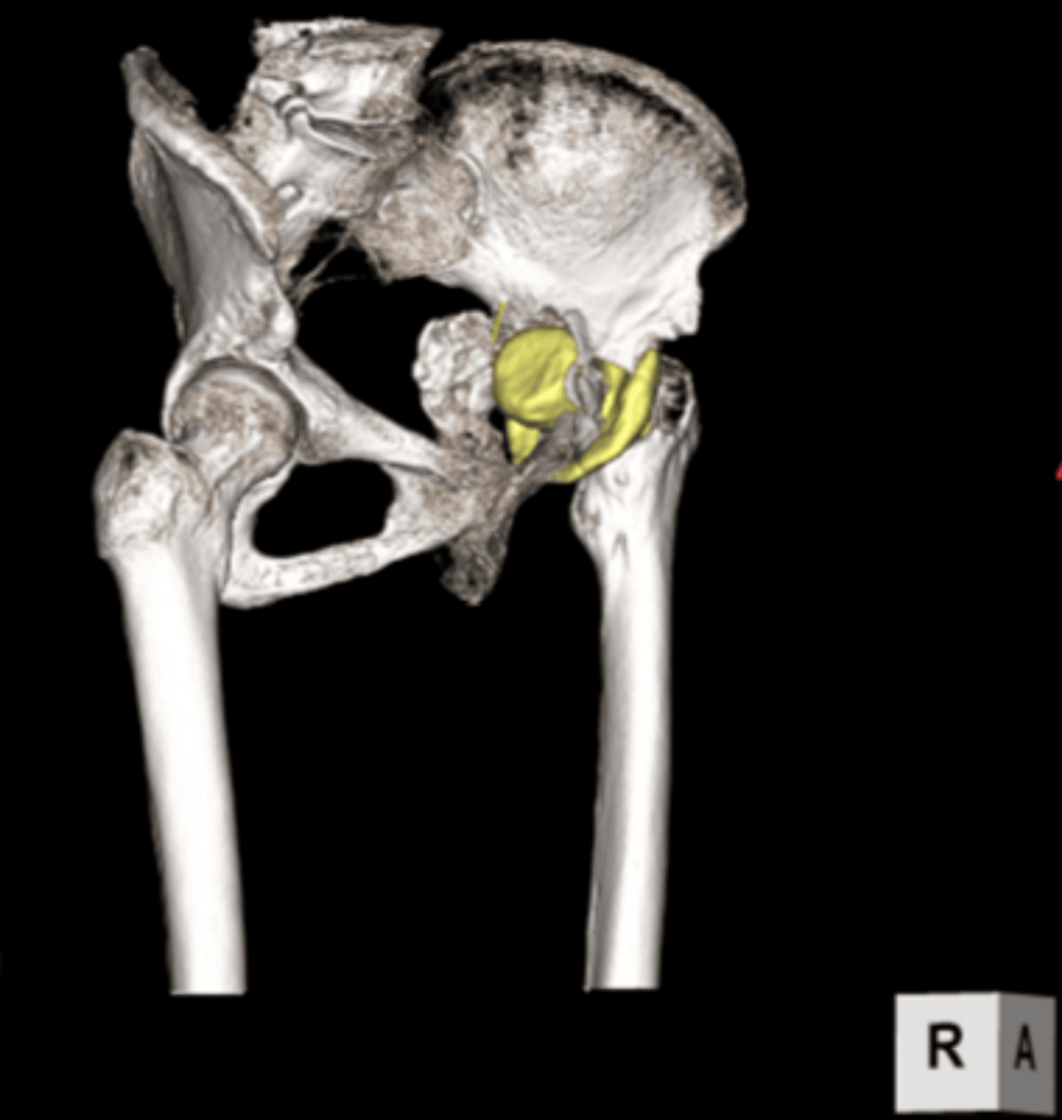
CT image of the hip after the first-stage removal The yellow region represents the cement spacer. Pelvic discontinuity with a large bone defect was observed.

The patient was transferred to our hospital on bed rest. Because there was a large bone defect in the pelvis (Figure 3), a staged reconstruction was scheduled. Ten days after primary THA, a first-stage removal operation was performed. This first-stage operation included spacer insertion and bone grafting on the fracture site, as well as implant removal (Figure 2C). After a two-week bed rest, ambulation without weight bearing (only toe touch permitted) was started. Rehabilitation with non-weight-bearing continued until the second-stage operation. Nine weeks after the first-stage operation, a second-stage reimplantation operation was performed. Substantial bone union was observed at the fracture site; therefore, a cementless cup with screws was used (Figure 2D). The operation time and blood loss were 144 min and 535 ml, respectively. Postoperative rehabilitation started with non-weight bearing, followed by half partial and full weight bearing at one and two weeks after the reimplantation, respectively. Two weeks after the reimplantation, PJI was suspected due to the wound and an elevated C-reactive protein of 9.69, and additional treatments, including debridement and continuous local antibiotic perfusion (CLAP), were added. Despite activity limitations due to CLAP and COVID-19 infection, the patient was able to walk independently with a walker and cane, two and three weeks after full weight-bearing, respectively. Nine weeks after the reimplantation, following the completion of intravenous antibiotics for PJI, the patient was discharged home with full independence in ADL.

Rehabilitation course

**Figure 4:**
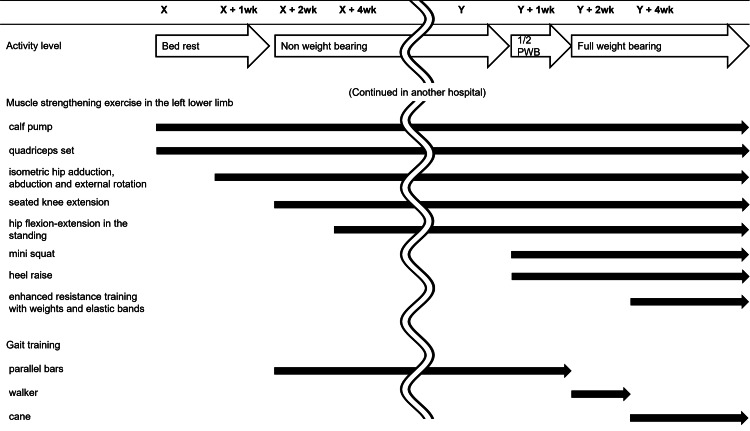
Description of the rehabilitation programs X, the day of the first-stage implant removal; Y, the day of the second-stage reimplantation

The rehabilitation course is illustrated in Figure 4. Postoperative rehabilitation was initiated on the day after the first-stage operation with on-bed exercise. In this phase, muscle strengthening exercises were focused on the unaffected regions to ensure bone healing. Specifically, only calf pumping and quadriceps sets were performed in the left lower limb, in addition to strengthening exercises using elastic bands in the unaffected limbs. One week later, isometric contractions of the hip muscles, such as adduction, abduction, and external rotation, were also started with close attention to the fracture site. Two weeks after the first-stage operation, ambulation with non-weight bearing was allowed; the patient started transferring to a wheelchair. The patient was soon able to transfer to a wheelchair with minimal assistance, and supervised gait training in parallel bars was initiated. At the same time, seated knee extension exercise was added, starting from active assistance and building up to manual resistance, depending on strength recovery. Active-assistive hip flexion-extension exercise in the standing position, approximately from 30-degree flexion to 0-degree extension, was also initiated at 4 weeks. Training exercises, as mentioned above, continued until the second-stage operation. Throughout the period, gait training was performed only in parallel bars because the patient was unable to walk with crutches.

On the day after the second-stage operation, ambulation without weight bearing was allowed. Exercises performed during the interim period, including gait training on parallel bars, were resumed. Furthermore, in contrast to the interim period, joint motion at the left hip improved due to reimplantation. Therefore, both muscle strengthening exercises, such as flexion and abduction, and passive ROM exercises in the left hip were enhanced. One week later, half partial weight-bearing was allowed. At that time, weight-bearing exercises, such as mini squats and heel raises, were started in addition to partial-weight gait training in parallel bars. A further week later, full weight bearing was permitted. At that time, since additional treatments for PJI were performed, only bedside exercise was feasible due to CLAP; nevertheless, exercises as closely resembling walking as possible were encouraged, such as single-leg standing and stepping exercises with one-handed support.

Upon completion of CLAP, the patient was able to walk independently with a walker. Unfortunately, however, the patient had contracted COVID-19 at that time and was quarantined in a ward. While the patient’s independent ambulation involved the use of a walker, supervised gait training with a cane was continued. A further week later, corresponding to five and three weeks after the second-stage operation and full weight-bearing, respectively, the patient was able to walk independently with a cane.

Change in physical function

The changes in physical function are presented in Table 1. Each parameter was measured based on the rest level at each time point. Knee extensor strength was measured using a handheld dynamometer with a restraining belt (μTas; ANIMA, Tokyo, Japan) in a seated position, with the knee at 60° flexion. Then, the torque (kgf*m) was calculated by multiplying the dynamometer value (kgf) and lever arm and was expressed as a percentage of the body weight (kgf*m/kg). Walking speed was measured over the middle 10 m of a 16-m walkway.

**Table 1:** Change in physical function

	Admission	X + 2wk	X + 4wk	X + 8wk	Y + 5wk	Y + 7wk	Y + 9wk
Handgrip strength (kgf; right/left)	13.0/11.3	15.7/15.1	14.0/12.2	14.8/15.2	15.3/12.8	15.3/14.6	14.3/13.5
Range of motion in the left (deg)							
Hip flexion	-	-	-	60	80	85	85
Hip abduction	-	-	-	5	15	15	15
Knee flexion	-	85	120	120	120	130	135
Knee extensor strength (kgf*m/kg; right/left)	-	-	0.058/0.027	0.081/0.043	0.083/0.047	0.093/0.049	0.090/0.049
Walking ability							
Comfortable speed (m/s)	-	-	-	-	0.53	0.63	0.71
Maximal speed (m/s)	-	-	-	-	0.67	0.75	0.90
Walking aid	-	-	-	-	Cane	Cane	Cane

During the first two weeks after the first-stage operation on bed rest, an improvement in upper limb strength was observed. Muscle weakness in the left lower limb was distinct at the time but improved throughout the interim period, expressed as knee extensor strength at four and eight weeks. After full weight-bearing was allowed after the second-stage operation, a gradual improvement in walking speed was observed.

## Discussion

In this report, we described a perioperative rehabilitation course for two-stage revision THA with acetabular fracture in a 75-year-old woman. Regardless of old age and long periods of non-weight-bearing, the patient was able to walk independently early after the reimplantation. Interim-period rehabilitation, including muscle-strengthening training, was assumed to have contributed to early postoperative gait acquisition without interfering with acetabular bone fusion. Our results suggest the safety and efficacy of both interim and postoperative rehabilitation for two-stage revision THA, even in patients with acetabular fractures.

Two-stage revision arthroplasty itself is not uncommon, as it is mainly performed for PJI. However, it often leads to poor functional outcomes [8,10,11], and rehabilitation strategies to improve these outcomes have not been established. Poor functional outcomes are assumed to be related to immobilization and disuse during the interim period, leading to decreased muscle function such as poor strength and flexibility. In this case, improvement in the muscle strength of the knee extensor on the affected limb, as well as unaffected limb strength, was observed during the interim period, suggesting the efficacy of muscle strengthening in both the affected and unaffected limbs. Therapists find it difficult to train the affected limb during the interim period because of limited joint motion due to the absence of an implant. The absence of implants may impede smooth joint movement and reduce the efficiency of muscle force transmission owing to joint instability. Therefore, instead of open kinetic chain exercises with a wide range of joint motion, isometric exercises with minimal joint motion of the hip muscles, especially the gluteal muscles, were used. These programs were successful in a case with an acetabular fracture, without interfering with bone healing, and therefore may apply to cases of generalized two-stage revision arthroplasty.

While muscle strengthening exercises are assumed to be successful, limited range of motion (ROM) remains a challenging issue. In this case, the final ROM of hip flexion remained at 85°. Soft-tissue contracture is recognized as one of the concerns in two-stage revision arthroplasty [5-8,10]. There seem to be two main causes of this problem during the interim period: joint immobilization and leg shortening [7,10,12,13]. Although passive stretching is commonly used to effectively improve soft tissue flexibility and joint ROM (i.e., to prevent contractures) [14-16], it is difficult to perform during prosthesis-free intervals in which joint motion is physically restricted. Alternative strategies to maintain soft tissue flexibility and joint ROM during the interim period are desirable.

Our results also provide insights into the rehabilitation protocol during CLAP for PJI. The patients experienced two weeks of activity restriction due to CLAP, in addition to nine weeks of prosthesis-free intervals. CLAP has become a novel treatment option for PJI [17,18]. Despite its superiority in infection resolution, the disadvantage is that the patient is restricted to mobility owing to the complicated route described previously [17]; bed rest is common during CLAP for safety reasons. In this case, however, although ambulation with mobility was still difficult, muscle strength training was continued via bedside weight-bearing exercises, and consequently, gradual recovery was observed. Exercise therapy during CLAP was thought to prevent disuse due to excessive rest and to contribute to functional recovery. Not only on-bed exercises but also weight-loaded exercises at the bedside could be an option for rehabilitation during CLAP.

## Conclusions

We reported a case of successful rehabilitation in two-stage revision THA for an intraoperative acetabular fracture. The interim-period rehabilitation, including muscle strengthening training, is assumed to have contributed to the early gait acquisition after reimplantation. Our results indicate the safety and effectiveness of interim-period rehabilitation in two-stage revision arthroplasty. Further studies with larger sample sizes are required to validate our findings.
